# Baseline D-dimer as a predictor of immune checkpoint inhibitor efficacy in cancer

**DOI:** 10.1080/07853890.2026.2620195

**Published:** 2026-01-28

**Authors:** Fan Li, Jie Zha, Meimei Hu, Wei Zhang

**Affiliations:** Department of Basic Medicine, Anqing Medical College, Anqing, Anhui, China

**Keywords:** Immune checkpoint inhibitors, D-dimer, cancer, overall survival, progression-free survival

## Abstract

**Background:**

Immune checkpoint inhibitors (ICIs) have revolutionized cancer treatment by enhancing antitumor immunity, yet durable responses are observed in only a fraction of patients. Identifying accessible and reliable biomarkers to predict therapeutic efficacy remains a critical unmet need. D-dimer, a fibrin degradation product reflecting systemic coagulation, has been associated with tumor progression and poor prognosis, but its predictive value in ICI-treated patients remains unclear.

**Methods:**

We conducted a systematic review and meta-analysis of studies evaluating baseline D-dimer in cancer patients receiving ICIs. Eligible studies reported outcomes including overall survival (OS), progression-free survival (PFS), objective response rate (ORR), or disease control rate (DCR). Data extraction and quality assessment were performed independently, and pooled hazard and odds ratios were calculated. Sensitivity and subgroup analyses were conducted to evaluate the stability of findings and potential cutoff-dependent effects.

**Results:**

Ten retrospective studies including 1,217 patients were analyzed. Elevated baseline D-dimer was significantly associated with worse OS (HR = 1.95, 95% CI: 1.62–2.36, *p* < 0.001) and shorter PFS (HR = 2.03, 95% CI: 1.57–2.63, *p* < 0.001). Higher D-dimer levels also correlated with lower ORR (OR = 0.42, 95% CI: 0.26–0.68, *p* < 0.001) and DCR (OR = 0.18, 95% CI: 0.10–0.34, *p* < 0.001). Sensitivity and subgroup analyses confirmed the robustness and consistency of these associations across tumor types, treatment regimens, and D-dimer thresholds.

**Conclusions:**

Baseline D-dimer is a readily measurable, cost-effective biomarker that predicts inferior outcomes in patients receiving ICIs. Its integration into clinical workflows may aid patient stratification and guide treatment decisions. Prospective multicenter studies are warranted to validate cutoff thresholds and further define its utility in optimizing immunotherapy efficacy.

## 1. Introduction

Cancer remains the leading cause of mortality worldwide and continues to exert an increasing burden on global health systems [1]. The introduction of monoclonal antibodies capable of blocking immune checkpoint pathways has dramatically transformed the therapeutic landscape of oncology [2,3]. Immune checkpoint inhibitors (ICIs)—particularly those targeting programmed cell death protein 1 (PD-1), programmed death-ligand 1 (PD-L1), and cytotoxic T-lymphocyte–associated protein 4 (CTLA-4)—have become foundational components of modern immuno-oncology [4,5]. By reinvigorating immune surveillance and amplifying antitumor immunity, these therapies have produced unprecedented clinical benefits across a diverse spectrum of malignancies [6]. Despite these advances, durable responses are observed in only a subset of patients, underscoring a critical need for robust predictive biomarkers that can guide patient selection and optimize therapeutic decision-making [7]. The identification of easily measurable, cost-effective, and reproducible biomarkers could expand the applicability of ICIs and enhance their overall clinical impact.

Decades of research have revealed a strong association between cancer biology and coagulation abnormalities. Hypercoagulable states and venous thromboembolism (VTE) are well recognized as contributors to tumor progression and poor prognosis across various malignancies [8,9]. Recent evidence highlights the prognostic value of circulating von Willebrand factor levels in metastatic melanoma patients treated with immune checkpoint blockade [10,11]. Moreover, ICI therapy itself appears to elevate the risk of VTE development, suggesting a complex interplay between immune modulation and coagulation cascades [9,12]. D-dimer, the terminal fibrin degradation product generated during fibrinolysis, is widely regarded as a sensitive indicator of coagulation activation and systemic thrombosis [13]. Elevated D-dimer concentrations have been consistently reported in gastric, pancreatic, and colorectal cancers compared with healthy controls and have shown positive correlations with metastatic burden [13,14]. These findings collectively suggest that D-dimer might serve as a candidate biomarker for monitoring tumor progression [15]. Nevertheless, whether baseline D-dimer levels are associated with the therapeutic efficacy of ICI-based regimens remains largely unexplored and lacks evidence-based consensus.

Although multiple inflammatory and coagulation-related biomarkers—such as C-reactive protein, neutrophil-to-lymphocyte ratio, platelet indices, and fibrinogen—have been investigated in the context of cancer prognosis, their specific relevance to immune checkpoint blockade remains inconsistent and often lacks mechanistic coherence. In contrast, D-dimer uniquely integrates information on both coagulation activation and fibrinolytic turnover, thereby reflecting a dynamic pathophysiological state that is closely intertwined with tumor-driven hypercoagulability, endothelial injury, and systemic inflammation. Importantly, emerging translational studies suggest that coagulation signaling may modulate antitumor immunity by influencing myeloid cell recruitment, cytokine release, and the formation of immunosuppressive fibrin-rich niches within the tumor microenvironment. These observations provide a biologically plausible rationale for investigating D-dimer, rather than a broad panel of coagulation markers, as a focused candidate biomarker for ICI-treated patients. However, the extent to which pretreatment D-dimer levels correlate with ICI responsiveness across malignancies remains uncertain, and no quantitative synthesis has yet clarified its predictive relevance. This gap underscores the need for a systematic evaluation specifically centered on baseline D-dimer as a potential determinant of immunotherapy outcomes.

Given the growing clinical use of ICIs and the urgent demand for accessible predictors of treatment response, clarifying the role of baseline D-dimer levels as a predictive biomarker is of considerable importance. A meta-analytic approach provides a rigorous method to address this knowledge gap by synthesizing results across heterogeneous studies, thereby increasing statistical power, minimizing random error, and improving the precision of effect estimates. Such an analysis allows for the assessment of consistency across cancer types, treatment regimens, and patient populations, enabling a more comprehensive understanding of the potential prognostic and predictive utility of D-dimer. The present meta-analysis was therefore undertaken to systematically evaluate the association between baseline D-dimer levels and the clinical outcomes of cancer patients treated with ICIs, with the ultimate aim of informing patient stratification and optimizing immunotherapy-based treatment strategies.

## 2. Methods

### 2.1. Literature search strategy

This meta-analysis was designed and executed in accordance with the PRISMA reporting guidelines [16]. A systematic search of PubMed, EMBASE, and the Cochrane Library was performed on August 23, 2025. The strategy incorporated both Medical Subject Headings (MeSH) and free-text terms to capture all relevant publications, including ‘Immune Checkpoint Inhibitors [MeSH]’, ‘PD-1 Inhibitors’, ‘PD-L1 Inhibitors’, ‘CTLA-4 Inhibitors’, and the names of commonly used ICIs such as ‘Pembrolizumab’, ‘Nivolumab’, ‘Atezolizumab’, ‘Ipilimumab’, ‘Avelumab’, ‘Tremelimumab’, ‘Durvalumab’, and ‘Cemiplimab’, as well as coagulation-related terms like ‘Fibrin Fragment D’ and ‘D-dimer’. Only articles published in English were considered eligible. The complete search algorithms for each database are provided in Supplementary material 1. To minimize publication bias, we additionally screened gray literature through Google Scholar and manually examined the reference lists of studies that met the inclusion criteria.

### 2.2. Inclusion and exclusion criteria

Studies were considered eligible if they satisfied all of the following conditions (1): participants had a confirmed diagnosis of malignancy (2); treatment regimens included ICIs (3); patients were categorized according to baseline D-dimer levels (high vs. low); and (4) at least one clinically meaningful outcome was reported, including overall survival (OS), progression-free survival (PFS), objective response rate (ORR), or disease control rate (DCR). Investigations were excluded when they consisted solely of meeting abstracts or opinion pieces. In instances where several articles analyzed overlapping populations, the report containing the most comprehensive data and methodologically robust design was selected for inclusion.

### 2.3. Data extraction and quality evaluation

For each eligible publication, essential study characteristics were systematically retrieved, including the first author’s name, year of appearance, study timeframe, country or region, tumor type, therapeutic regimen, cohort size, patient demographics (age and sex), and the D-dimer cutoff value applied. When hazard ratios (HRs) were reported, multivariable-adjusted estimates were prioritized over those derived from univariable analyses whenever possible [17].

The methodological rigor of all observational cohorts was evaluated using the Newcastle–Ottawa Scale (NOS), which appraises three key domains. The first domain, Selection (maximum of four points), examines cohort representativeness, adequacy of the comparison group, accuracy of exposure ascertainment, and confirmation that participants were outcome-free at baseline. The second domain, Comparability, awards up to two points based on design or analytic control for confounding. The final domain, Outcome, grants a maximum of three points by assessing outcome measurement, follow-up length, and completeness of follow-up. Studies achieving more than six points were classified as high quality. Data abstraction and quality scoring were performed independently by two reviewers, and any disagreements were resolved in consultation with the senior investigator.

### 2.4. Statistical analysis

All meta-analyses were conducted using Stata software, version 18.0. Pooled results were visually represented by forest plots. Between-study heterogeneity was evaluated with both Cochran’s Q test and the I^2^ statistic, with heterogeneity considered meaningful when I^2^ exceeded 50% or when Q test p-values were below 0.1. In the presence of substantial heterogeneity, pooled estimates were calculated using the DerSimonian–Laird random-effects model; otherwise, a fixed-effect approach based on the Inverse Variance method was applied [17].

Potential publication bias was examined with Begg’s rank correlation and Egger’s regression tests. To evaluate result stability, leave-one-out sensitivity analyses were performed, sequentially excluding each study and recalculating the combined HRs. In addition, subgroup analyses were carried out by stratifying studies according to the cancer types, Cox model, and D-dimer cutoff thresholds. A two-sided *p*-value <0.05 was interpreted as statistically significant.

## 3. Results

### 3.1. Search results and study characteristics

**Figure 1.**
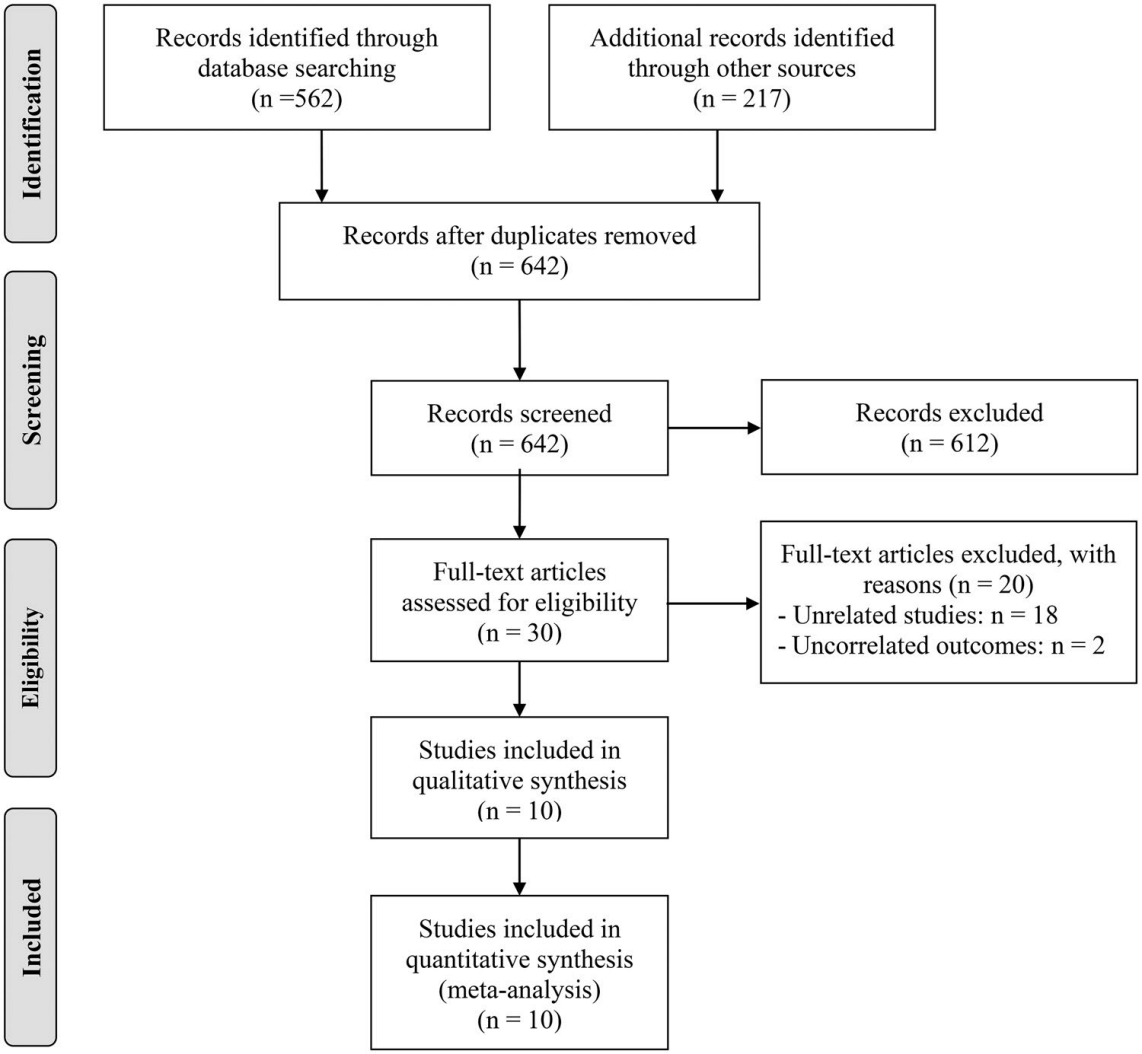
Flowchart depicting the selection process of eligible studies.

A systematic search of the selected databases, complemented by manual screening of reference lists, initially identified 779 candidate records. After eliminating 137 duplicates, 612 reports were excluded based on title and abstract screening because they failed to satisfy the predefined eligibility criteria. Full-text review of the remaining 30 publications led to the exclusion of 20 studies that did not meet inclusion standards. Ultimately, 10 studies were considered eligible and were incorporated into the quantitative synthesis (Figure 1) [10,18–26].

Key study characteristics are summarized in Table 1. Altogether, 1,217 participants were analyzed, with individual sample sizes ranging from 40 to 277. Eight studies originated from China and two from Germany. The tumor types represented included three cohorts with non–small-cell lung cancer, and one cohort each with gastric carcinoma, lung adenocarcinoma, cutaneous squamous cell carcinoma, esophageal carcinoma, and melanoma. All investigations followed a retrospective observational design. Methodological quality, as assessed using the Newcastle–Ottawa Scale (NOS), ranged between 6 and 8 points, reflecting an overall low risk of bias (Table 1).

**Table 1.** Main characteristics of the studies included.

Study	Country	Cancer type	Treatment	Study period	Sample size	Age	Gender (male/female)	Cut‐point	NOS
Xu et al. 2025	04/2021-03/2023	Gastric cancer	China	PD-1/PD-L1 inhibitors	40	20/20	15/25	Optimal cutoff value (0.97 μg/mL)	6
Li et al. 2025	01/2019-12/2023	Lung adenocarcinoma	China	ICIs	102	76/26	65 (35–78)^a^	Normal reference value (0-0.5 mg/L)	7
Geidel et al. 2025	11/2019-12/2024	Cutaneous squamous cell carcinoma	Germany	Cemiplimab	45	33/12	78 (55 − 90)^b^	Optimal cutoff value (0.91 μg/mL)	6
Wu et al. 2024	07/2017-05/2021	Esophageal cancer	China	PD-1/PD-L1 inhibitors	233	215/18	61.89 ± 7.71	Optimal cutoff value (0.24 μg/mL)	7
Wang et al. 2024	04/2019-05/2023	Small-cell lung cancer	China	PD-1/PD-L1 inhibitors	137	100/37	61^c^	Optimal cutoff value (0.68 μg/mL)	7
Pang et al. 2024	01/2018-05/2024	Non-small-cell lung cancer	China	PD-1 inhibitors	100	84/16	65 (40–84)^a^		6
Hu et al. 2023	02/2019-01/2023	Small-cell lung cancer	China	Durvalumab	100	89/11	63 (56–71)^b^	Optimal cutoff value (0.88 μg/mL)	6
Li et al. 2022	01/2015-03/2019	Non-small-cell lung cancer	China	PD-1/PD-L1 inhibitors	277	213/64	61 (33–91)^a^	Normal reference value (0-0.5 mg/L)	7
Chen et al. 2023	01/2018-02/2020	Non-small-cell lung cancer	China	PD-1 inhibitors	100	84/16	–	Optimal cutoff value (0.98 μg/mL)	7
Stadler et al. 2023	03/2018-02/2022	Melanoma	Germany	ICIs	83	55/28	64.95 ± 15.70	Normal reference value (0.2–0.5 mg/L)	6

^a^ Median (range).

^b^ Median (IQR).

^c^ Median.

ICIs: immune checkpoint inhibitors; PD-1: Programmed Death Receptor 1; PD-L1: Programmed Cell Death Ligand 1.

### 3.2. Baseline D-dimer and overall survival

**Figure 2.**
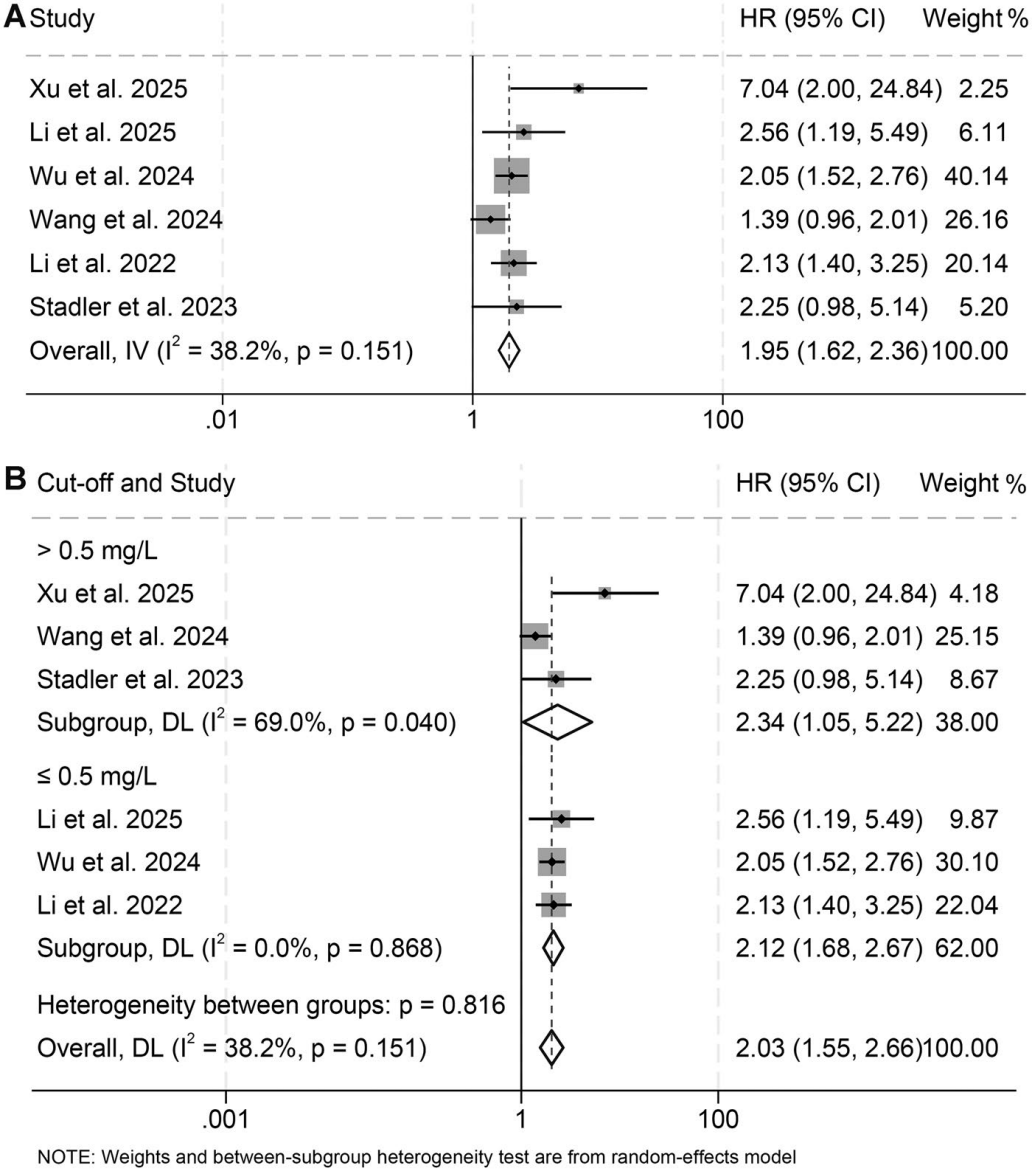
Forest plot summarizing the link between pretreatment D-dimer levels and overall survival among cancer patients receiving immune checkpoint inhibitors (A). Stratified analyses using different cutoff thresholds further delineated this association (B). HR: hazard ratio; CI: confidence interval.

Six eligible studies comprising 872 participants were included to evaluate the prognostic impact of baseline D-dimer on OS in patients receiving ICIs. Pooled HR demonstrated that elevated D-dimer levels were significantly correlated with inferior OS (HR = 1.95, 95% CI: 1.62–2.36, *p* < 0.001; Figure 2A). Heterogeneity across studies was minimal, as indicated by Cochran’s Q test and an I^2^ statistic of 38.2% (*p* = 0.151), justifying the application of a fixed-effects model. Stratified analyses based on D-dimer cutoff values (≤0.5 vs. >0.5), cancer types, and Cox model yielded consistent results, further supporting the robustness of this association (Figure 2B and Figure S1).

**Figure 3.**
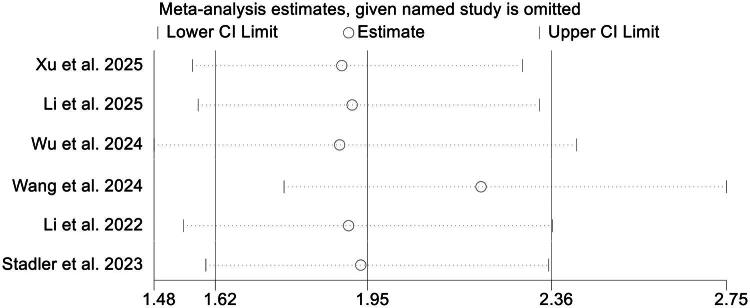
Sensitivity assessment evaluating the relationship between pretreatment D-dimer concentrations and overall survival in cancer patients receiving immune checkpoint inhibitors. CI: confidence interval.

Sensitivity analyses, performed by sequentially omitting individual studies, showed no substantial fluctuation in the combined HR, reinforcing the stability of the findings (Figure 3). Moreover, evaluations for publication bias using Begg’s and Egger’s tests did not reveal significant asymmetry (Begg’s *p* = 0.260; Egger’s *p* = 0.160), suggesting that small-study effects were unlikely to have distorted the overall outcome.

### 3.3. Pretreatment D-dimer and progression-free survival

**Figure 4.**
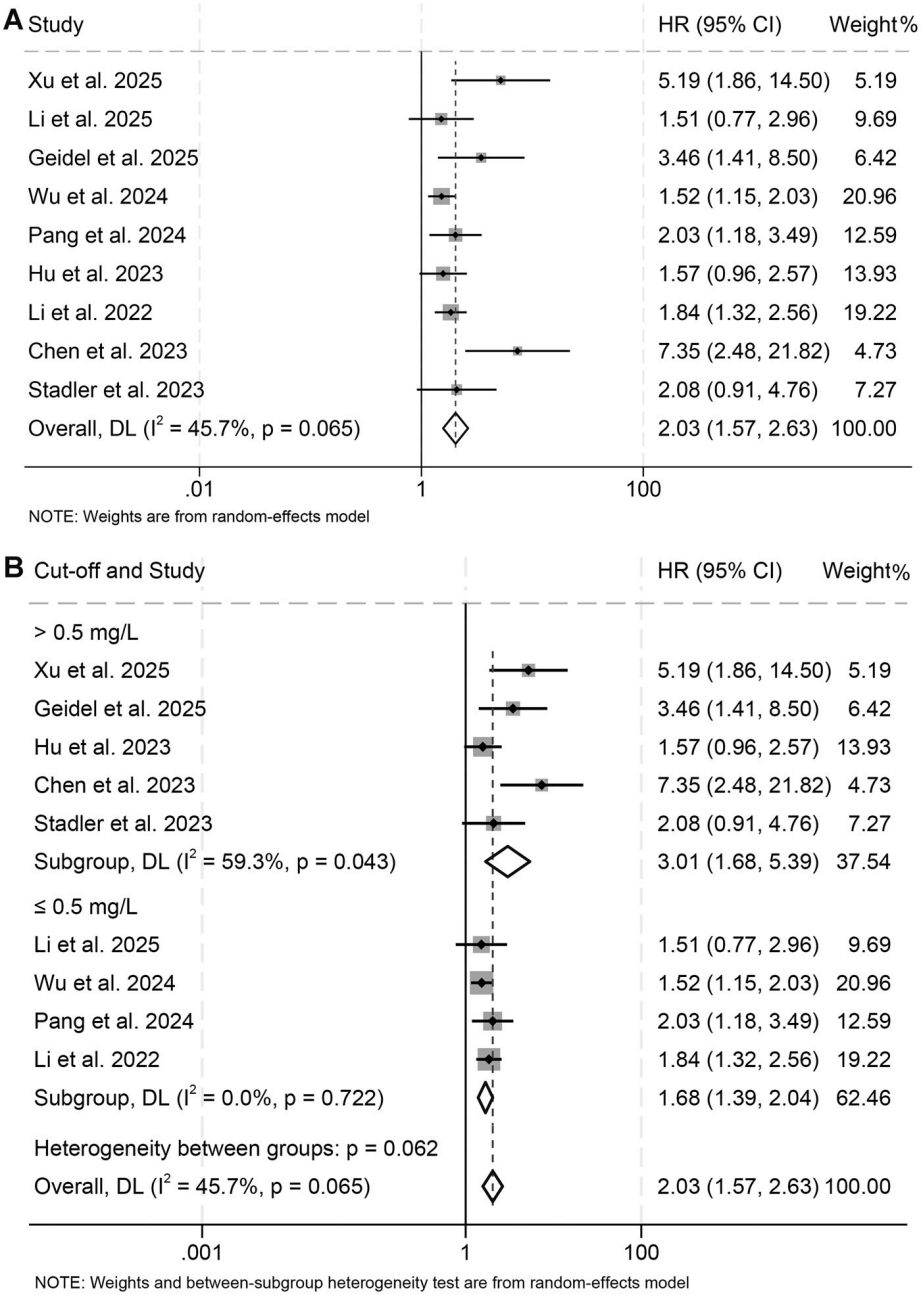
Forest plot summarizing the link between pretreatment D-dimer levels and progression-free survival among cancer patients receiving immune checkpoint inhibitors (A). Stratified analyses using different cutoff thresholds further delineated this association (B). HR: hazard ratio; CI: confidence interval.

Nine studies involving 1,080 participants were analyzed to determine the prognostic significance of pretreatment D-dimer levels for PFS in patients receiving ICIs. The combined HR demonstrated that elevated D-dimer levels were strongly associated with shorter PFS (HR = 2.03, 95% CI: 1.57–2.63, *p* < 0.001; Figure 4A). Moderate heterogeneity was detected across studies (I^2^ = 45.7%, *p* = 0.065), and therefore a random-effects model was applied. Subgroup analyses stratified by cutoff values (≤0.5 *vs.* >0.5) and cancer types produced concordant findings, reinforcing the consistency of the observed relationship (Figure 4B and Figures S2, S3).

**Figure 5.**
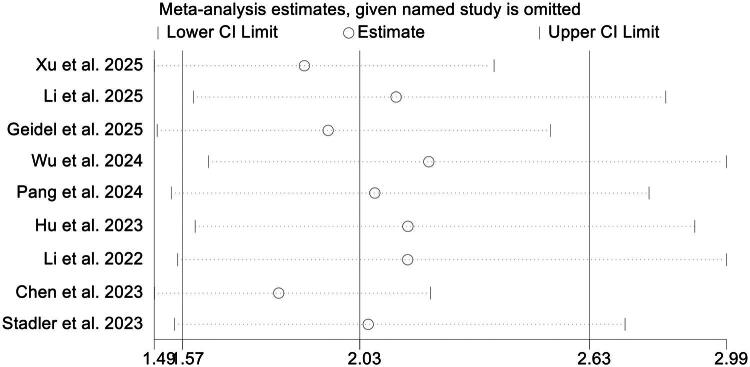
Sensitivity assessment evaluating the relationship between pretreatment D-dimer concentrations and progression-free survival in cancer patients receiving immune checkpoint inhibitors. CI, confidence interval.

Sequential sensitivity analyses, in which each study was removed one at a time, demonstrated that the overall pooled estimates remained largely unchanged, underscoring the robustness of the results (Figure 5). Moreover, Begg’s and Egger’s statistical tests for publication bias showed no significant asymmetry (Begg’s *p* = 0.105; Egger’s *p* = 0.112), suggesting that the findings were unlikely to be influenced by small-study effects.

### 3.4. D-dimer and objective response rate

**Figure 6.**
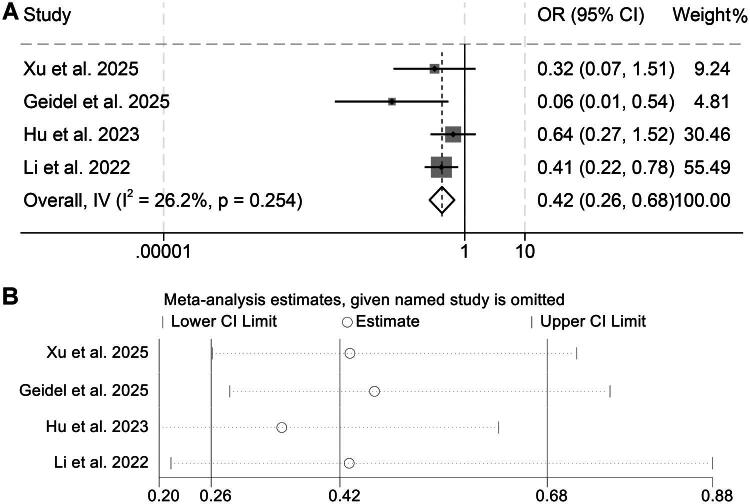
Forest plot summarizing the link between pretreatment D-dimer levels and objective response rate among cancer patients receiving immune checkpoint inhibitors (A). Sensitivity analysis of the association between baseline D-dimer and objective response rate in cancer patients treated with immune checkpoint inhibitors (B). OR: odds ratio; CI: confidence interval.

To further clarify the relationship between D-dimer levels and ORR, we analyzed four eligible studies encompassing 462 patients with malignancies. Because heterogeneity among these studies was minimal (I^2^ = 26.2%, *p* = 0.254), a fixed-effects approach was adopted. Pooled results indicated that individuals with higher baseline D-dimer concentrations experienced markedly reduced ORR compared with those exhibiting lower levels (OR = 0.42, 95% CI: 0.26–0.68, *p* < 0.001; Figure 6A).

The reliability of this association was verified by sensitivity analyses, in which the stepwise exclusion of each study yielded consistent overall estimates (Figure 6B). Additionally, Begg’s and Egger’s statistical assessments found no significant evidence of publication bias (Begg’s *p* = 0.308; Egger’s *p* = 0.295), supporting the robustness of the observed association.

### 3.5. D-dimer and disease control rate

**Figure 7.**
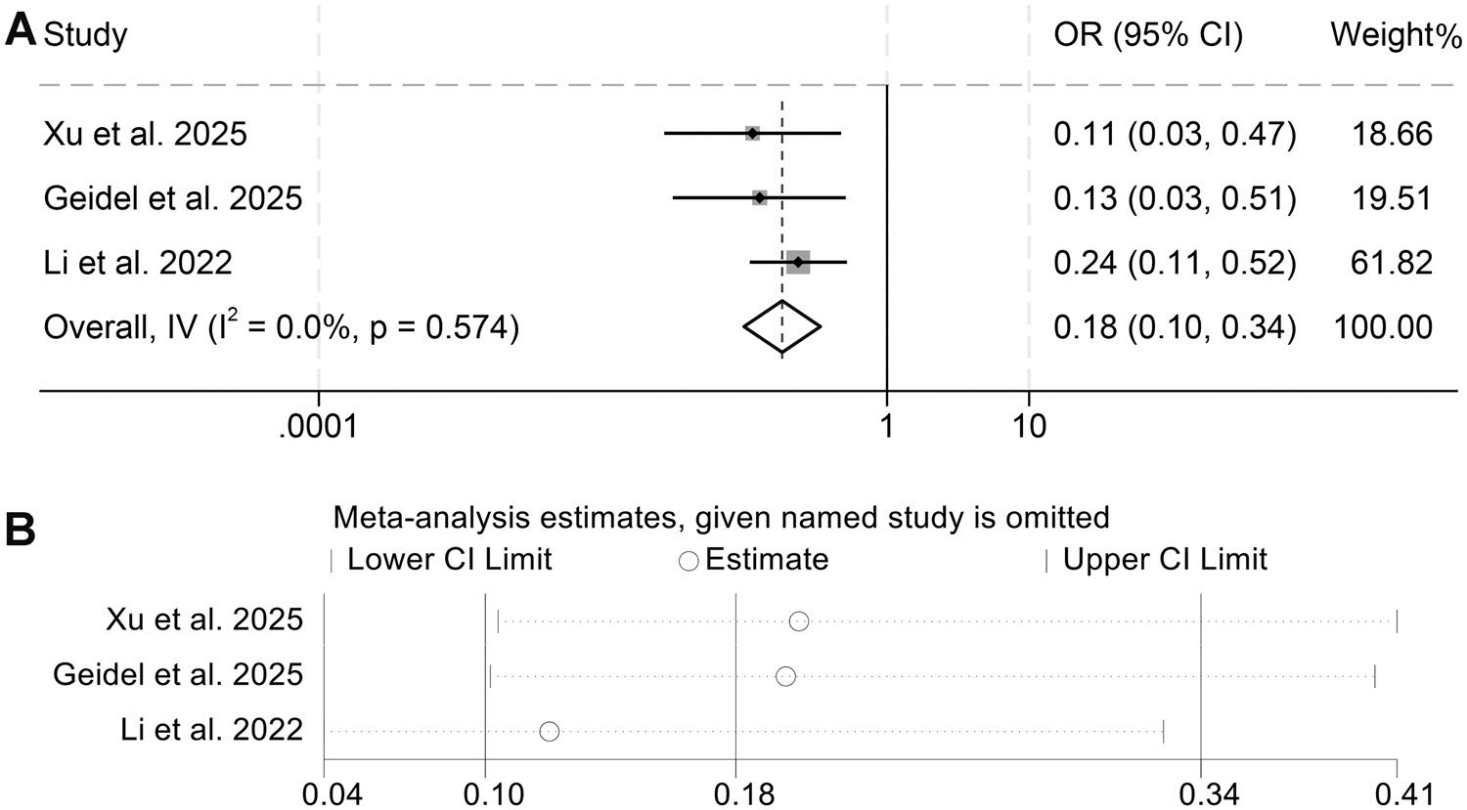
Forest plot summarizing the link between pretreatment D-dimer levels and disease control rate among cancer patients receiving immune checkpoint inhibitors (A). Sensitivity analysis of the association between baseline D-dimer and objective response rate disease control rate in cancer patients treated with immune checkpoint inhibitors (B). OR: odds ratio; CI: confidence interval.

The association between baseline D-dimer levels and DCR was evaluated using data from three independent studies including 362 patients. Because no meaningful heterogeneity was detected across these datasets (I^2^ = 0, *p* = 0.574), a fixed-effect model was applied for pooled estimation. The combined analysis indicated that individuals with elevated D-dimer concentrations exhibited a markedly reduced probability of achieving disease control relative to those with lower levels (OR = 0.18, 95% CI: 0.10–0.34, *p* < 0.001; Figure 7A).

To ensure the robustness of this observation, we conducted sensitivity analyses by sequentially removing each contributing study. The stability of the effect size across all iterations reinforced the reliability of the overall findings for DCR (Figure 7B).

## 4. Discussion

D-dimer, a readily quantifiable marker of fibrin turnover, offers a cost-effective and widely accessible means to assess systemic coagulation activity. Our meta-analysis revealed that elevated baseline D-dimer levels were consistently associated with inferior survival outcomes in patients with various malignancies. This observation suggests that the coagulation milieu before the initiation of immune checkpoint blockade may critically influence therapeutic efficacy. These results are consistent with accumulating evidence linking hypercoagulability and systemic inflammation to tumor progression, immune evasion, and reduced responsiveness to anticancer therapy [27,28]. Consequently, baseline D-dimer may serve as a pragmatic blood-based biomarker to stratify patients, inform therapeutic choices, and identify those who may benefit from closer monitoring or adjunctive treatment during ICI-based regimens.

Mechanistic insights from preclinical and translational research provide further biological plausibility. Systemic activation of coagulation cascades is frequently observed in patients with advanced malignancies, resulting in fibrin deposition and microthrombus formation [27–29]. Coagulation mediators—including tissue factor (TF), factor VIIa (FVIIa), factor Xa (FXa), and thrombin—have been shown to facilitate immune escape through distinct pathways. TF–FVIIa signaling enhances PD-L1 expression *via* PAR2 activation in murine breast cancer models [30], and elevated TF expression has been correlated with poor prognosis and diminished immune effector infiltration in triple-negative breast cancer (TNBC) [31]. Similarly, coagulation factor Xa (FXa)–PAR2 signaling suppresses CD8^+^ T-cell infiltration and cytokine secretion within tumors [32], while thrombin contributes to immune evasion by activating platelets, releasing TGF-β1, and inhibiting NK-cell activity [33,34]. The thrombin/PAR-1 pathway has also been implicated in attenuating antitumor immunity in pancreatic ductal adenocarcinoma [35]. Collectively, these mechanisms create a prothrombotic, immunosuppressive tumor microenvironment that shields circulating tumor cells (CTCs) from cytotoxic immune cells and may diminish the efficacy of ICIs.

Emerging evidence further strengthens the biological rationale linking Emerging evidence further strengthens the biological rationale linking hypercoagulability to impaired ICI efficacy by demonstrating that coagulation proteases can directly modulate immune-checkpoint expression. A recent mechanistic study in hepatocellular carcinoma showed that FXa activates PAR-2 signaling to induce anoikis resistance and immune escape, and—critically—upregulates PD-L1 transcription through STAT2 phosphorylation [36]. FXa-activated PAR-2 signaling reduced CD8^+^ T-cell infiltration and cytokine secretion, while pharmacologic FXa inhibition downregulated tumor PD-L1 expression and restored antitumor immunity [36]. Moreover, combining the FXa inhibitor rivaroxaban with anti-PD-1 therapy produced synergistic antitumor effects *in vivo* and improved response rates in clinical observations. These findings indicate that coagulation-driven PAR signaling can directly enhance PD-L1–mediated immune evasion, supporting the concept that elevated D-dimer may reflect not only tumor burden or systemic inflammation but also a pro-coagulant molecular program that actively promotes resistance to immune checkpoint blockade [36]. Nonetheless, current evidence remains limited regarding the regulation of PD-1 or CTLA-4 expression by coagulation factors, and future translational studies are warranted to define the full spectrum of coagulation–immune checkpoint interactions.

Moreover, fibrin-rich microthrombi formed on the surface of malignant cells have been reported to act as physical barriers, limiting immune recognition and clearance by natural killer cells [37]. We further hypothesize that such barriers may sterically hinder immune checkpoint receptor engagement, thereby impairing T-cell activation and diminishing the antitumor effects of PD-1/PD-L1 blockade. These insights underscore the need to integrate coagulation status into immunotherapy response prediction models and highlight the potential of D-dimer as a clinically actionable biomarker.

Despite the robustness of our findings, several limitations should be acknowledged. All included studies were retrospective in design, which introduces potential selection bias and unmeasured confounding. An additional methodological challenge is the substantial variability in D-dimer cut-off definitions across studies. Because each cohort applied its own threshold to classify high versus low D-dimer levels, direct comparability of biomarker categorization was limited, introducing potential measurement bias. To accommodate this, we adopted the ≤0.5 vs >0.5 mg/L categories based on the distribution of reported values rather than standardized clinical criteria; consequently, these subgroup analyses should be viewed as exploratory given their restricted statistical power.

Another important limitation is the limited ability to account for differences in treatment regimens. Many patients received ICIs as part of combination therapy, yet most studies did not provide sufficient detail about concomitant anticancer agents or their relative contributions to clinical outcomes. As a result, we could not stratify analyses by monotherapy versus combination therapy. Although the consistent association between elevated D-dimer and poorer outcomes across cancer types—and across both univariable and multivariable Cox models—suggests that D-dimer may have prognostic relevance beyond treatment composition, we cannot exclude the possibility that D-dimer also reflects responsiveness to accompanying agents. Future studies with detailed treatment stratification are needed to clarify this issue.

Our capacity to further investigate sources of heterogeneity was also limited. Although heterogeneity was minimal for most outcomes, the moderate variability in PFS could not be fully explained because key clinical modifiers—such as ICI type, line of therapy, and regimen composition—were insufficiently reported. The small number of eligible studies further precluded meta-regression or more granular subgroup analyses beyond tumor type. Additionally, because all included cohorts originated from China or Germany, the generalizability of our findings to broader populations may be restricted.

These methodological constraints highlight the need for prospective, multicenter studies employing standardized biomarker thresholds, comprehensive reporting of treatment characteristics, and harmonized data-collection frameworks. Such efforts will help clarify whether coagulation status serves as a prognostic marker specifically for ICI response or more broadly reflects overall treatment resistance. Looking ahead, our findings also raise the intriguing possibility that targeting hypercoagulability may enhance the effectiveness of immune checkpoint blockade. Future trials evaluating anticoagulation strategies in combination with ICIs could provide important insights into optimizing therapeutic outcomes.

In addition to these limitations, several strengths of our work merit emphasis. To our knowledge, this study represents the most comprehensive synthesis to date examining the prognostic relevance of baseline D-dimer specifically in patients treated with immune checkpoint inhibitors across multiple tumor types. By integrating data from heterogeneous clinical settings and conducting a series of robust sensitivity, subgroup, and Cox model–stratified analyses, we were able to consistently demonstrate the direction and magnitude of the association between elevated D-dimer and inferior outcomes. The uniformity of these findings—despite differences in cancer types, treatment contexts, and analytical approaches—suggests that D-dimer captures a fundamental biological process relevant to immunotherapy responsiveness. Moreover, our work addresses an important and previously unresolved clinical question by systematically evaluating a widely available, inexpensive, and routinely measured biomarker that could be readily implemented in real-world practice. In this regard, the present meta-analysis not only consolidates the existing evidence base but also provides a conceptual and methodological foundation for future prospective studies aimed at validating D-dimer as a practical tool for patient risk stratification and treatment optimization in immuno-oncology.

## 5. Conclusion

Our findings provide compelling evidence that baseline D-dimer levels are closely linked to clinical outcomes in patients undergoing ICI therapy. Elevated pretreatment D-dimer consistently predicted inferior OS, shorter PFS, and lower response rates in malignancies. These results underscore the value of D-dimer as an inexpensive, reproducible, and clinically accessible biomarker that could facilitate patient stratification and guide therapeutic decision-making in immuno-oncology. Future prospective, multicenter studies employing standardized cutoff thresholds are warranted to validate these observations and to clarify whether integrating coagulation-based metrics into treatment algorithms can optimize the efficacy of ICI-based regimens.

## Supplementary Material

Supplementary material.docx

PRISMA 2020 Checklist.docx

## Data Availability

The datasets generated and analyzed during the current study can be obtained from the corresponding author upon justified request.
