# Supplementary material for: Baseline D-dimer as a predictor of immune checkpoint inhibitor efficacy in cancer
Source: Ann Med. 2026 Jan 28;58(1):2620195. doi: 10.1080/07853890.2026.2620195 (PMC12857687; doi:10.1080/07853890.2026.2620195)
Supplement: Supplementary material.docx [file IANN_A_2620195_SM6745.docx]

Supplementary material 1. Detailed article search strategy

((Camrelizumab) OR (Sintilimab) OR (Tislelizumab) OR (Toripalimab) OR (Envafolimab) OR (Immune Checkpoint Inhibitors) OR (Checkpoint Inhibitors, Immune) OR (Immune Checkpoint Inhibitor) OR (Checkpoint Inhibitor, Immune) OR (Immune Checkpoint Blockers) OR (Checkpoint Blockers, Immune) OR (Immune Checkpoint Blockade) OR (Checkpoint Blockade, Immune) OR (Immune Checkpoint Inhibition) OR (Checkpoint Inhibition, Immune) OR (PD-L1 Inhibitors) OR (PD L1 Inhibitors) OR (PD-L1 Inhibitor) OR (PD L1 Inhibitor) OR (Programmed Death-Ligand 1 Inhibitors) OR (Programmed Death Ligand 1 Inhibitors) OR (PD-1-PD-L1 Blockade) OR (Blockade, PD-1-PD-L1) OR (PD 1 PD L1 Blockade) OR (CTLA-4 Inhibitors) OR (CTLA 4 Inhibitors) OR (CTLA-4 Inhibitor) OR (CTLA 4 Inhibitor) OR (Cytotoxic T-Lymphocyte-Associated Protein 4 Inhibitors) OR (Cytotoxic T Lymphocyte Associated Protein 4 Inhibitors) OR (Cytotoxic T-Lymphocyte-Associated Protein 4 Inhibitor) OR (Cytotoxic T Lymphocyte Associated Protein 4 Inhibitor) OR (PD-1 Inhibitors) OR (PD-1 Inhibitor) OR (PD 1 Inhibitors) OR (Inhibitor, PD-1) OR (PD 1 Inhibitor) OR (Programmed Cell Death Protein 1 Inhibitor) OR (Programmed Cell Death Protein 1 Inhibitors) OR (Pembrolizumab) OR (Nivolumab) OR (Atezolizumab) OR (Ipilimumab) OR (Avelumab) OR (Tremelimumab) OR (Durvalumab) OR (Cemiplimab) OR (Immune Checkpoint Inhibitors[MeSH Terms])) AND ((D-dimer) OR ("fibrin fragment D" [Supplementary Concept]))
